# The effect of Internet use on nutritional intake and health outcomes: new evidence from rural China

**DOI:** 10.3389/fnut.2024.1364612

**Published:** 2024-04-08

**Authors:** Zhilong Deng, Jian Liu, Yu Hong, Weigang Liu

**Affiliations:** ^1^School of Information Engineering, Shaanxi Xueqian Normal University, Xi’an, China; ^2^Leibniz Institute of Agricultural Development in Transition Economies (IAMO), Halle, Germany; ^3^Institute of Agricultural Economics and Development, Chinese Academy of Agricultural Sciences, Beijing, China; ^4^Department of Agricultural Economics, Kiel University, Kiel, Germany

**Keywords:** Internet use, food consumption behaviors, dietary quality, nutrition outcomes, chronic diseases

## Abstract

**Introduction:**

Internet use is changing nutritional intake and health outcomes, but the results are mixed, and less attention is given to the rural developing regions. Based on the China Health and Nutrition Survey (CHNS) data from 2004 to 2015, this study seeks to better understand the effect of Internet use on nutritional intake and health outcomes.

**Methods:**

An instrumental variable estimation is used to address endogeneity problem.

**Results:**

The results show that Internet use improves the dietary knowledge of rural residents, and thus has a positive impact on dietary quality, such as healthy eating index (HEI) and dietary diversity score (DDS). The higher the dietary quality, the better the nutritional health status. However, results also show that Internet use increases the risk of overweight, and obesity among rural Chinese residents. Because Internet use has significantly reduced the physical activity of rural residents in China. Interestingly, we also find that the Internet increases the risk of chronic diseases such as diabetes and high blood pressure, but there is a positive causal relationship between Internet use and the self-assessment score of health.

**Discussion:**

Our findings suggest that there may be a serious lack of awareness of the health risks of chronic diseases among Chinese rural residents. Therefore, policymakers are suggested to consider the possible negative effects when promoting digital development.

## 1 Introduction

In the past four decades, the Chinese dietary pattern has undergone significant changes, mainly characterized by higher intakes of fats, added caloric sweeteners, animal source foods as well as a higher reliance on processed foods (1–3). These changes have important consequences. First, increasing food consumption could improve the nutrition status of Chinese residents. According to estimates from the Food and Agriculture Organization of the United Nations (FAO), the prevalence of malnutrition in China has dropped from 24% in the 1990s to less than 10% (1). However, the overconsumption of high-calorie foods and processed foods is associated with increased rates of non-communicable diseases such as overweight, obesity, and diabetes. It is reported that more than half of Chinese residents are overweight or obese, with the overweight and obese rates being 34.3 and 16.4%, respectively (4). Compared to urban areas in China, rural areas have lower incomes, poor infrastructure, and often limited access to food and markets. As a result, overweight and obesity have become a major public health problem, but about 150.8 million people in China are still malnourished or micronutrient deficient (5, 6). Thus, it is important to understand the drivers of diet transition and nutrition outcomes.

Existing studies have mainly discussed the effects of income and urbanization on nutrient intake and health outcomes, while little attention has been paid to the role of Internet use. The literature finds that income elasticity of animal products (such as milk and its products, meat, and aquatic products) is high, while that of staple foods such as whole grains and starches is small or even negative (1). As a result, the diets of low-income people tend to be dominated by staple foods, which are the most cost-effective source of calories. As incomes rise, consumers increasingly focus on other attributes of food in addition to nutrition, such as appearance, taste, status value, odor, and degree of processing (7). Healthy foods generally cost more, and low-income people are more likely to settle for poor health choices in developed countries (8). Study also found that income affects health through five channels: nutritional intake, dietary diversity, dietary knowledge, food preferences, and eating out (6, 8). Urbanization may also affect nutritional transition and health. This is because people in cities are often closer to markets and have a wider choice of food (6). In addition, in the process of urbanization, individuals with different food cultural backgrounds influence each other (9). This interaction may lead to a shift in food preferences, so the nutritional transition also changes with the development of cities.

Internet use may also affect nutritional intake and health outcomes, but results are mixed and there is a lack of research on rural areas in developing countries. First, Internet use can improve food accessibility by allowing residents to access food that is not available in nearby supermarkets (10). Second, the Internet can reduce the transaction costs in the agricultural market and promote farmers’ market participation, thereby improving consumers’ affordability of food. More importantly, the Internet is the main channel for accessing food and nutrition information, and Internet use can further improve consumer dietary knowledge (11). These changes can further improve the dietary quality of rural residents. Based on the household survey data of 10,042 rural households in six provinces in China, Xue et al. (12) found that Internet use can increase calorie, protein and fat intake, thereby improving the nutritional status of rural residents. Shen et al. found that the dietary diversity of Internet users is higher than the respondents who do not use the Internet (10). The results of Ma and Jin’s (13) study showed that Internet use can significantly improve the dietary quality of rural residents in China. And one possible channel is that Internet use improves rural residents’ dietary knowledge, thereby optimizing their dietary structure. Cui et al. (14) found that Internet use increases dietary quality by cultivating consumers’ more positive attitudes toward diet. Similar results have been confirmed in other countries, such as Kenya and Australia (15, 16). These studies confirm that Internet use improves dietary quality in rural areas of China. Higher dietary quality means better nutritional health (17, 18). In addition, Internet can also serve as a way to promote healthy eating habits and increase physical activity interventions to reduce the risk of chronic diseases such as obesity and high blood pressure (19).

**Figure 1:**
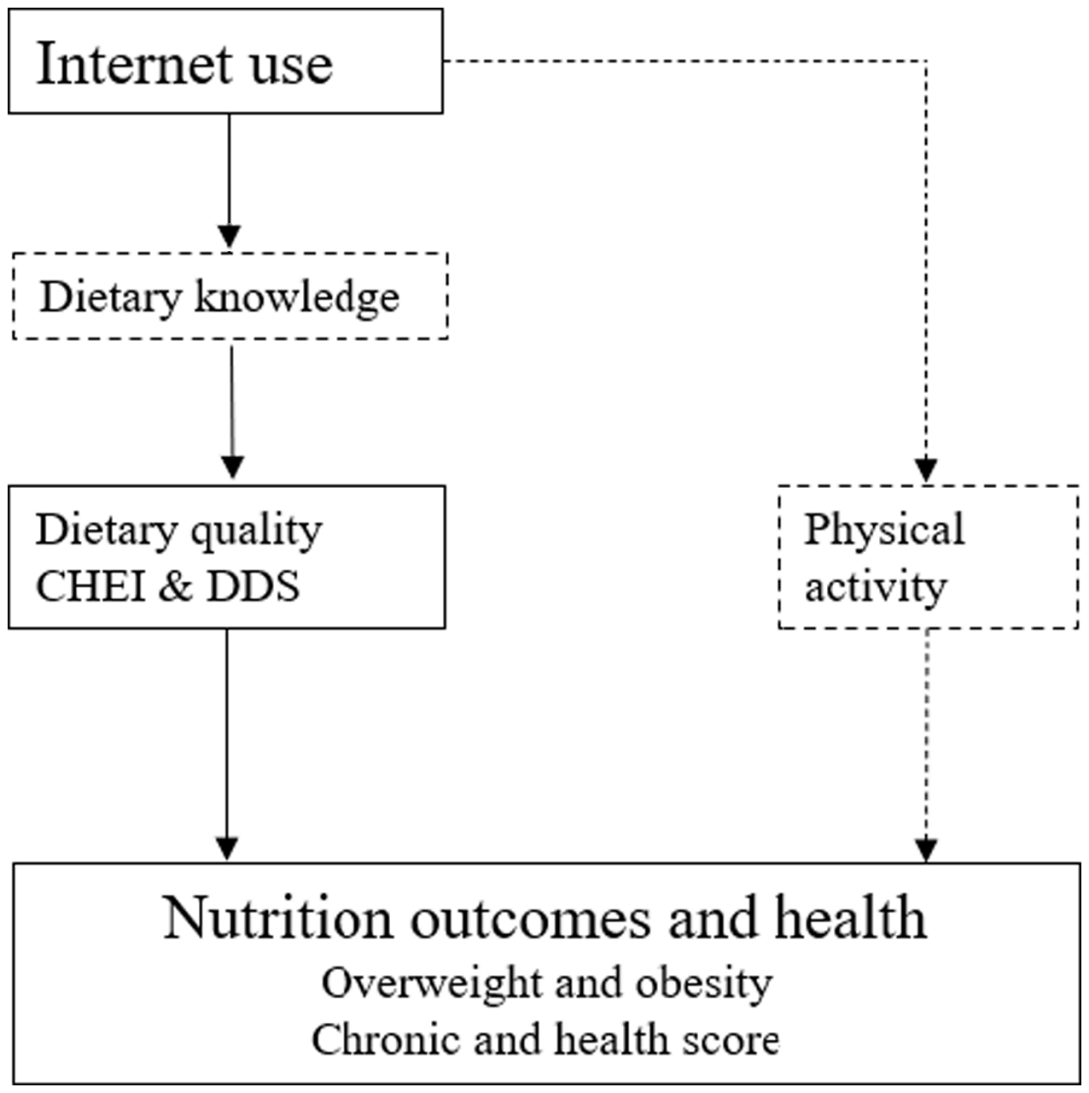
Theoretical analysis framework.

**Figure 2:**
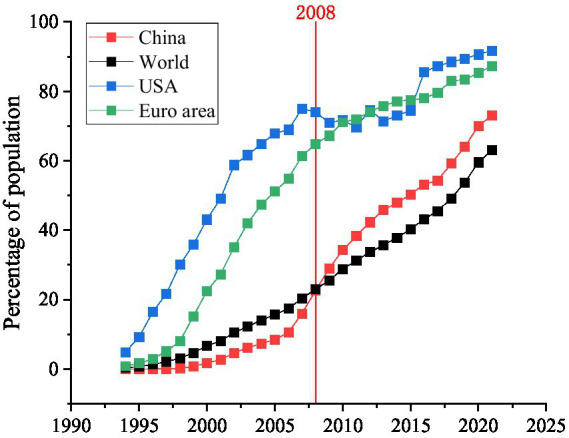
Individuals using the Internet as a percentage of the total population in China and the world from 1994 to 2021. Data source: The World Bank (https://data.worldbank.org/).

However, Internet use also have a negative impact on nutrition and health. Other studies have suggested that Internet use may increase the risk of overweight and obesity by increasing sedentary lifestyles and decreasing the amount of time spent outside (20). Internet addiction, especially among adolescents, can significantly affect eating behavior and nutritional health. Specifically, Internet-addictive users had higher rates of irregular bedtimes and smoking and alcohol abuse than non-addictive Internet users. In addition, among high-risk Internet users, irregular dietary behaviors due to loss of appetite, frequent skipping meals, and snacking may lead to unbalanced nutrient intake and health problems (21). Physical activity is considered to be an important way to maintain caloric balance and health. However, excessive use of Internet time may reduce the time spent on physical activity. According to the 51st China Internet Development Statistics Report, Chinese netizens spend an average of 3.8 h online every day. It should be noted that the majority of time spent online in rural China is spent watching videos and playing video games (22). Therefore, there may be a substitution effect between Internet use and physical activities. In other words, Internet use may reduce the physical activity of rural residents and therefore have negative effects on nutrition and health. The theoretical framework of how Internet use affects nutritional intake and health outcomes is shown in Figure 1. In the past decades, China’s Internet penetration has been on an upward trend, and by the end of 2016, the number of Internet users in China reached 731 million, ranking first in the world (13). According to the China Internet Network Information Center (CNNIC), there will be nearly 1.051 billion Internet users in China by 2022, and the Internet penetration rate will reach 74.4%. Figure 2 shows the trend of Internet development in China and the world. As shown in Figure 2, the number of Internet users in China is the first in the world, but compared with many European and American companies, there is still great room for development. Therefore, the effects of Internet use on nutritional intake and health outcomes may be long-lasting, and it is important to understand how Internet use affect nutritional intake and health outcomes in rural China.

The objective of the study is to better understand the effect of Internet use on nutritional intakes and health outcomes and shed light on its underlying channels. To this end, using CHNS panel data from 2004 to 2015, we employ regression models to quantify the effects and use instrument methods to solve the potential endogeneity problem.

This study contributes to the existing literature in the following three ways. First, unlike the previous studies focusing on the influences of urban residents’ Internet use on nutritional health, this study expands the content of existing studies in rural areas. Second, extant literature only focuses on the causal relationship between Internet use and nutritional health, without exploring the influencing mechanism. Therefore, the second contribution of this study is to provide a more detailed discussion of how Internet use may influence nutritional intake and health outcomes through two potential channels: diet quality and physical activity. Third, studies have extensively been done in developed world, while Internet use and nutritional health in most developing nations received few attentions. The results of this study not only have important reference value for the sustainable development of rural areas in China, but also have important reference value for the formulation of food policies and digital economy development systems in developing countries such as India, Vietnam and Thailand.

The reminders of this study as follows. The second part introduces CHNS data and methods of this study. The third part describes the empirical results. The fourth part compares the results with the literature and analyzes the limitations of this study. The last part is the conclusion.

## 2 Materials and methods

### 2.1 Data

This study uses data from the CHNS, which covers 10 waves (1989, 1991, 1993, 1997, 2000, 2004, 2006, 2009, 2011, and 2015) in 12 provinces (Guangxi, Guizhou, Henan, Hubei, Heilongjiang, Hunan, Jiangsu, Liaoning, Shandong, Shaanxi, Yunnan, and Zhejiang) and 3 autonomous cities (Beijing, Chongqing, and Shanghai). A multistage random cluster design is used to select the CHNS sample. Specifically, one high-income city and another low-income city are first selected from each province. Following this sample strategy, CHNS selected two counties in each city, two urban communities, and two rural villages in each county. In each community and village, 20 households are randomly selected for participation. In this process, it included approximately 7,200 households and over 30,000 individuals as part of its longitudinal dataset. Note that, CHNS includes a large number of questions about target family members and their food consumption, nutrition, and health-related questions, and it is one of the most widely used data for nutrition and health-related research (23–25).

To fulfill the purpose of this study, two criteria are applied to the CHNS dataset. First, we only use a sample of respondents whose age equals and/or larger than 18 years old living in rural areas. Second, as the information on food consumption and dietary knowledge is only available from 2004 to 2011, so, we used the respondents from 2004 to 2011 for food consumption analysis and respondents from 2004 to 2015 for nutrition outcomes and health analysis. Finally, 27,390 observations and 11,292 individuals in 167 villages are obtained for analysis. Because this study uses unbalanced panel data, there are small differences in the sample size of regression models with different dependent variables.

### 2.2 Variables

#### 2.2.1 Internet use

The dependent variable is Internet use. This is a dummy variable defined as 1 if the respondent uses the Internet and 0 otherwise.

#### 2.2.2 Nutritional intake and health outcomes

In this study, the healthy eating index (HEI) for Chinese and the dietary diversity score (DDS) is used as the proxy variables of nutritional intake. HEI is designed as a scoring system to measure dietary quality and is recently developed by Yuan et al. (26) According to the Dietary Guidelines for Chinese (DGC) in 2016, all food groups are divided into adequacy and limitation components. Adequate components include 12 food groups (total grains, whole grains and mixed beans, tubers, vegetables, dark vegetables, fruits, dairy, soybeans, fish and seafood, poultry, eggs, and seed and nuts) and limitation ingredients include 5 food groups (red meat, cooking oils, sodium, added sugars, and alcohol). The DGC gave the maximum and minimum daily recommendations for the adequate and limitation food groups, respectively. Then all food groups are weighted to a maximum of 5 or 10 scores and HEI is the total score between 0 and 100. Higher HEI scores indicate better dietary health of respondents. For more details on the establishment of the HEI, please see Supplementary Table S1. In this study, due to the lack of information on daily intake of added sugars, the highest score for HEI is 95. As shown in Table 1, the mean value of HEI is 60.1 which is comparable to some literature (6, 26).

**Table 1:** The descriptive statistics of the main variables.

Variables		Pooled	Internet use	Non-Internet use	Diff.
Dependent variables					
HEI	Healthy eating index for Chinese	60.647 (6.813)	62.100 (7.308)	60.408 (6.706)	1.692***
DDS	Dietary diversity score	4.824 (3.129)	4.366 (3.461)	4.896 (3.052)	−0.530***
Overweight	= 1 if overweight; = 0 otherwise	0.289 (0.453)	0.364 (0.481)	0.284 (0.451)	0.081***
Obesity	= 1 if obesity; = 0 otherwise	0.062 (0.242)	0.096 (0.294)	0.060 (0.238)	0.036***
Chronic	= 1 if chronic disease; = 0 otherwise	0.022 (0.145)	0.011 (0.102)	0.023 (0.149)	−0.012***
Health score	Self-assessment score of health	4.956 (2.284)	5.544 (2.611)	4.917 (2.256)	0.627***
Channel variables					
Knowledge	Dietary knowledge	6.117 (3.653)	7.821 (3.132)	5.842 (3.656)	1.979***
Activities	Physical activity	2.339 (2.883)	0.378 (0.990)	2.470 (2.920)	−2.092***
Control variables					
Age	Age of the respondent (years)	42.412 (15.525)	35.942 (12.647)	42.843 (15.603)	−6.902***
Marriage	= 1 if married; = 0 otherwise	0.817 (0.398)	0.795 (0.404)	0.819 (0.398)	−0.024***
Education	Education of the respondent (years)	7.518 (4.183)	12.257 (4.321)	7.194 (3.972)	5.062***
Work	= 1 if able to work; = 0 otherwise	0.860 (0.347)	0.884 (0.321)	0.859 (0.348)	0.025***
Income	Household income	8.836 (1.067)	9.930 (0.943)	8.763 (1.035)	1.167***
Family size	Number of family members	20.109 (28.541)	36.551 (46.882)	19.010 (26.512)	17.541*** 0.081

Diff is the *t*-test results and ***p < 0.01; **p < 0.05; *p < 0.1. The standard deviation is in parentheses.

The dietary diversity score (DDS) is also widely used to calculate the dietary quality (27) and it can be calculated as Equations 1 2.


(1) $$DDS_{i}=\{\begin{array}{c} 1,\;\mathrm{if}\;x_{i}>0\\ 0,\;\mathrm{if}\;x_{i}=0 \end{array}$$

$$\mathrm{Where}\;x_{i}=\left\{\begin{array}{c} \mathrm{Total}\;\mathrm{Grains};\;\mathrm{Tubers};\;\mathrm{Vegetables};\;\mathrm{Fruits};\;\mathrm{Dairy};\;\mathrm{Soybeans};\\ \mathrm{Fish}\;\mathrm{and}\;\mathrm{Seafood};\;\mathrm{Poultry};\;\mathrm{Red}\;\mathrm{Meat};\;\mathrm{Eggs};\;\mathrm{Seed}\;\mathrm{and}\;\mathrm{Nuts} \end{array}\;\right\}$$

(2) $$DDS=\overset{10}{\underset{I=1}{\sum }}DDS_{i}$$

where $x_{i}$ is the daily food intake of food group i; the value of $DDS_{i}$ is 1 if the respondent has eaten the i-th food in the last 3 days of the survey, otherwise $DDS_{i}$ is 0. Finally, the DDS is the sum of all the food groups ranging between 0 and 10. As is shown in Table 1, the mean value of DDS is 4.824 which is similar to Shen et al. (10). For more details on the consumption of each food group, please refer to Supplementary Figure S1.

**Figure 3:**
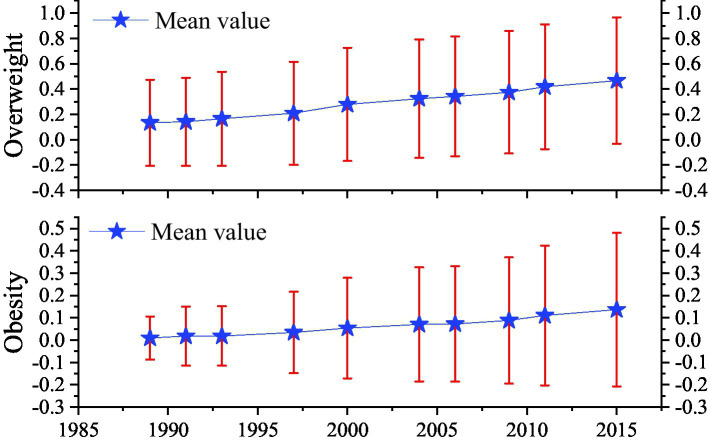
The trend of nutrition outcomes from 1989 to 2015 in rural China. The upper and lower end points are the mean plus or minus the standard deviation. Source: Authors’ calculation based on the CHNS data from 1989 to 2015.

Four dependent variables are used in this study to measure health outcomes: overweight, obesity, self-assessment score of health and chronic disease. The CHNS dataset contains the height and weight of the respondents and can be used to calculate the respondents’ body mass index (BMI), which is weight divided by the square of height (in kg/m^2^). Following the existing literatures, the respondents with a BMI equal to or greater than 24 are defined as overweight, and equal to or greater than 28 are defined as obesity (4, 28). The results in Table 1 show that the main values of overweight and obesity between 2004 and 2015 in rural China are 28.9 and 6.2%, which are similar to the findings of Ren et al. (29). In addition, as can be seen from Figure 3, both the mean and standard deviation of overweight and obesity in rural China show an increasing trend. This means the obesity problem in rural China is becoming more and more serious, and the difference in obesity among residents is also increasing.

The self-assessment score of health is also widely used by many researchers (30–32). In the CHNS survey, one of the questions is how healthy do you think you are? The respondents have to choose a number from 1 to 10. However, many researchers argue that this variable is too subjective to obtain accurate health status information (30, 33, 34). Thus, we also use whether the respondents had a chronic disease (diagnosed with high blood pressure and diabetes) as our measure of health variable. Indeed, we also believe that the second objective variable is more reasonable. However, in this study, we can further discuss whether the self-assessment score of health is a reliable variable to measure health status by comparing the regression results of the two dependent variables.

#### 2.2.3 Dietary knowledge and physical activities

Regarding the channel variable of dietary knowledge, it is calculated on 12 questions on basic dietary knowledge. Referring to the criteria recommended by the WHO and the study by Ren et al. (6). We constructed a brief index based on the respondents’ answers: the correct answer is assigned a value of 1 and the wrong answer is assigned a value of −1, so the dietary knowledge ranges from −12 to 12. This measure has been widely used to assess an individual’s dietary knowledge, and more details are presented in Supplementary Table S2. A higher score indicates greater knowledge of food consumption and nutritional intake. As shown in Table 1, the average score of dietary knowledge is 6.117, and the average score of dietary knowledge in the Internet use sample is 1.979 higher than that in the non-Internet use sample.

The CHNS data also tracked the physical activities of the respondents. Specifically. CHNS data recorded respondents’ participation in sports in the past week, such as martial arts (Kung Fu, etc.), gymnastics, dance, acrobatics, track and field (running, etc.), swimming, football, basketball, tennis, badminton, volleyball, etc. In this study, the physical activity variable is the number of types of activities that respondents had participated in in the past week. As is shown in Table 1 that the physical activities of Internet users are significantly lower than that of non-Internet users.

#### 2.2.4 Control variables

The control variables in this study include age, marriage, education, work status, income, and household size. Usually, young people tend to use the Internet more than older people. As shown in Table 1, the average age of Internet users is 35.9 which is well lower than the non-Internet users of 42.8 years. We also controlled for the household income and respondents’ level of education. First, there is a certain cost associated with using the Internet, considering the relatively lower income levels of the rural population in China. As income increases, the likelihood of using the Internet may correspondingly increase. Secondly, most rural Chinese are exposed to the Internet later, and individuals with higher levels of education may be more inclined to learn and use the Internet. In addition, we also controlled the work status, marriage status, and household size. More detailed descriptive statistics of the main variables are presented in Table 1.

### 2.3 Methods

To examine the effect of Internet use on food consumption and health in rural China, this study used the following benchmark model:


(3) $$Y_{\mathrm{it}}=\alpha_{0}+\alpha_{1}\mathrm{Interne}t_{\mathrm{it}}+\alpha_{2}X_{\mathrm{it}}+\varepsilon_{\mathrm{it}}$$

Where $Y_{\mathrm{it}}$ is the dependent variables such as HEI, DDS, and health score; Internet is the independent variable and $\alpha_{1}$ is the average treatment effect of Internet use on food consumption and health; $X_{\mathrm{it}}$ is a series of control variable like age, marital status, education, work status, household income, and family size; and the $\varepsilon_{\mathrm{it}}$ is the error term.

In this study, three key dependent variables of overweight, obesity and chronic disease are binary variables. To investigate the relationship between Internet use and these dummy variables, we start with the benchmark model as Equation 4.


(4) $$\mathrm{Pr}\left(Z_{\mathrm{it}}=1\;|\;\mathrm{Interne}t_{\mathrm{it}},\;X\right)=\theta_{0}+\theta_{1}\mathrm{Interne}t_{\mathrm{it}}+\theta_{2}X_{\mathrm{it}}+\varepsilon_{\mathrm{it}}$$

Where the dependent variable $Z_{\mathrm{it}}$ include overweight, obesity and chronic disease; $\theta_{1}$ is the change in the probability of using Internet; Other variables are the same as in Equation 3.

When the endogeneity problem is not considered, the estimated coefficient of $\alpha_{1}$ and $\theta_{1}$ are unbiased and consistent. However, the respondents can self-select whether to use the Internet or not. In other words, the sample is not random. Besides, some unobserved variables that have changed over the years could also affect food consumption and health. These problems may lead to bias and inconsistencies in estimated results.

To solve these problems, we applied the instrumental variable estimation to calculate the “net” effect of Internet use on food consumption and health, as shown in Equations 5 and 6. Similarly, the instrumental variable estimation for overweight, obesity and chronic disease are shown in Equation 5 and 7.


(5) $$\mathrm{Interne}t_{\mathrm{it}}=\beta_{0}+\beta_{1}IV_{\mathrm{it}}+\beta_{2}X_{\mathrm{it}}+\tau_{\mathrm{it}}$$

(6) $$Y_{\mathrm{it}}=\gamma_{0}+\gamma_{1}{\hat{\mathrm{Internet}}}_{\mathrm{it}}+\gamma_{2}X_{\mathrm{it}}+\varphi_{\mathrm{it}}$$

(7) $$\mathrm{Pr}\left(Z_{\mathrm{it}}=1\;|\;\mathrm{Interne}t_{\mathrm{it}},\;X\right)=\tau_{0}+\tau_{1}{\hat{\mathrm{Internet}}}_{\mathrm{it}}+\tau_{2}X_{\mathrm{it}}+\sigma_{\mathrm{it}}$$

Where $IV_{\mathrm{it}}$ is the instrumental variable of Internet use; $X_{\mathrm{it}}$ is several control variables; ${\hat{\mathrm{Internet}}}_{\mathrm{it}}$ is the predicted value of $\mathrm{Interne}t_{\mathrm{it}}$ ; $\gamma_{1}$ is the net effect of Internet use on food consumption and health; $\tau_{1}$ is the change in the probability of using Internet; Here, $\gamma_{1}$ and $\tau_{1}$ are unbiased and consistent.

In this study, we take the proportion of residents using the Internet to the total village residents as the instrumental variable. This instrumental variable is expected as valid mainly for two reasons. First, it has a direct effect on the Internet use. Because the residents live close to each other in rural China, there is a replication effect for Internet use among neighbors (35). Second, it is exogenous to food consumption and health at the individual level, because it can influence their food consumption and health only by affecting their Internet use. Note that, this instrumental variable has been widely used in previous studies (36, 37). In addition, the Hausman test is used to confirm whether the variable of Internet use is endogenous and F-test is applied to test the weak IV problems. If the Hausman test rejects the null hypothesis, then the IV estimation is better and if the value of F statistic greater than 10, it indicates there is no weak IV problems in this study.

## 3 Results

### 3.1 The effect of Internet use on nutritional intake and health outcomes

The IV estimation results for the effect of Internet use on nutritional intake and health outcomes are presented in Table 2. The *p* value of the Hausman test is less than 0.001 and all the F statistic values are greater than 10, which indicates that IV estimation strategies are necessary and the hypothesis of weak instrumental variables is rejected. The results with and without control variables are consistent, which further indicates that our estimates are robust.

**Table 2:** The effect of Internet use on nutritional intake and health outcomes.

	HEI	HEI	DDS	DDS
Internet use	6.071*** (18.259)	1.987*** (5.606)	7.616*** (49.688)	0.572*** (8.511)
Control variables	No	Yes	No	Yes
Wald chi^2^	1270.14***	8488.57***	333.40***	1758.30***
Observations	20,679	20,105	20,679	20,105
	Overweight	Overweight	Obesity	Obesity
Internet use	1.368*** (30.049)	0.263*** (3.885)	1.177*** (23.531)	0.196** (2.048)
Control variables	No	Yes	No	Yes
Wald chi^2^	902.97***	5890.29***	553.72***	2343.38***
Observations	26,077	25,366	26,077	25,366
	Chronic	Chronic	Health score	Health score
Internet use	0.286*** (3.319)	0.512*** (3.618)	2.150*** (24.554)	2.556*** (19.356)
Control variables	No	Yes	No	Yes
Wald chi^2^	11.01***	837.52***	637.66***	5709.91***
Observations	27,079	26,485	27,380	26,563

The fixed effects are used to estimate the results and the robust standard error deviation is in parentheses. ***p < 0.01; **p < 0.05; *p < 0.1.

As is shown in the upper of Table 2, Internet use can significantly increase HEI and DDS. Specifically, the mean values of HEI and DDS of Internet users are about 1.987 and 0.572 higher, respectively, than those of non-Internet users. The estimated coefficients of results with control variables are larger than those without control variables, suggesting that the results may be overestimated if control variables are not taken into account. These results suggest that Internet use can improve dietary quality.

The middle part of Table 2 is the estimated results for health outcomes. The coefficients of overweight and obesity are all positively significant at 1% level, which means the Internet could increase the overweight and obesity risk among adults in rural China. The coefficients of chronic are also positive and significantly at 1% level, which suggests that respondents who use the Internet may have higher rates of high blood pressure and diabetes. Regression estimates of overweight, obesity, and chronic disease results suggest that Internet use may be harmful to health. However, the estimated effect of health scores indicates a positive relationship between Internet use and the self-assessment score of health, which may be due to a serious lack of awareness of the health risks of chronic diseases among Chinese rural residents.

### 3.2 The effect of Internet use on dietary knowledge and physical activity

The estimated results of dietary knowledge and physical activity are presented in Table 3. First, when the control variables are included, the estimated coefficient of dietary knowledge is 2.008 and is significant at the 1% level, indicating that Internet use can increase respondents’ dietary knowledge. Generally speaking, the higher the dietary knowledge, the higher the level of dietary quality. Therefore, Internet use can improve dietary quality by improving dietary knowledge. Second, our estimated coefficient of physical activity is -0.117 and significant at the 1% level, indicating that Internet use significantly reduces respondents’ physical activity, which further negatively impact nutritional outcomes and health. Our results suggest that the positive effects of Internet use may be smaller than the negative effects in rural China. Therefore, the overall impact of Internet use on nutrition outcomes and the health of rural residents is negative. In particular, Internet use increases the likelihood of overweight, obesity, high blood pressure, and diabetes.

**Table 3:** The effect of Internet use on dietary knowledge and physical activity.

	Knowledge	Knowledge	Activities	Activities
Internet use	6.758*** (41.185)	2.008*** (12.689)	−1.658*** (−35.639)	−0.117*** (−2.752)
Control variables	No	Yes	No	Yes
Wald chi^2^	1696.18***	28215.73***	1270.14***	8479.18***
Observations	26,617	26,100	27,390	26,563

The fixed effects are used to estimate the results and the robust standard error deviation is in parentheses. ***p < 0.01; **p < 0.05; *p < 0.1.

### 3.3 Heterogeneity analysis

This section focuses on the heterogeneity of Internet use on nutritional intake and health outcomes across gender, age, and income. These results have been considered control covariates. First, we checked whether the baseline results of this study also apply to the male and female samples, and the specific estimates are shown in Table 4. The results show that Internet use has a significant positive effect on HEI and DDS in both male and female samples, which is consistent with our baseline regression results. However, there are clear gender differences in the effect of Internet use on overweight and obesity. Specifically, Internet use significantly increased the probability of overweight and obesity for the male sample but had no significant effect on the female sample. Regression results for the two health variables showed that there is a positive relationship between Internet use and chronic disease and the Self-assessment score of health in both male and female samples, but the estimated coefficient of Internet use for chronic disease in males is not significant.

**Table 4:** The heterogeneity analysis by gender.

	HEI	DDS	Overweight	Obesity	Chronic	Health score
Male sample						
Internet use	6.193*** (10.923)	0.917*** (9.467)	0.414*** (4.526)	0.238* (1.717)	0.350 (1.606)	2.580*** (14.418)
Wald chi^2^	2097.67***	181193.44***	3538.68***	1124.26***	375.39***	2821.52***
Observations	9,975	13,378	12,638	12,638	13,339	13,378
Female sample						
Internet use	7.170*** (11.811)	1.109*** (10.065)	0.062 (0.629)	0.087 (0.613)	0.674*** (2.966)	2.465*** (12.678)
Wald chi^2^	2241.70***	167359.59***	2566.24***	1090.06***	378.77***	3021.18***
Observations	10,130	13,185	12,728	12,728	13,146	13,185

The fixed effects are used to estimate the results and the robust standard error deviation is in parentheses. ***p < 0.01; **p < 0.05; *p < 0.1.

The second heterogeneity analysis strategy of this study is to analyze the differences in Internet use on nutritional intake and health outcomes across different age groups. As proposed by WHO, it defines young people with age below 44 years old; middle-aged and elderly people are those with age over 45 years old. So, we defined those aged below 45 as the youth group and those aged 45 and above as the elderly group, with its respective results shown in Table 5. The estimated coefficients of the aged group are significantly positive and larger than those of the young group. Thus, Internet use is likely to have a greater impact on nutrition and health in the elderly age group than in the younger age group. Besides, in the youth group, the estimated coefficients of HEI, DDS and Health score are significantly positive, while the estimated coefficients of overweight, obesity and Chronic are not statistically significant.

**Table 5:** The heterogeneity analysis by age.

	HEI	DDS	Overweight	Obesity	Chronic	Health score
Sample: Age < 45						
Internet use	1.001** (2.119)	1.092*** (9.501)	−0.151 (−0.996)	0.034 (0.346)	−0.884 (−1.605)	2.928*** (15.320)
Wald chi^2^	624.62***	137921.09***	2777.10***	1044.53***	70.80***	3314.28***
Observations	8,281	10,512	9,779	9,779	6,810	10,512
Sample: Age ≥ 45						
Internet use	8.600*** (11.719)	1.812*** (12.031)	0.709*** (4.100)	0.767*** (6.142)	1.109*** (5.046)	3.566*** (12.522)
Wald chi^2^	2460.51***	211548.79***	2783.07***	974.64***	471.38***	2774.74***
Observations	11,824	16,051	15,587	15,587	15,998	16,051

The fixed effects are used to estimate the results and the robust standard error deviation is in parentheses. ***p < 0.01; **p < 0.05; *p < 0.1.

As mentioned above, farmers with higher incomes are more likely to have access to Internet. In this study, we defined the top 50 percent of the sample as a high-income group and the bottom 50 percent as a low-income group. The regressions of Internet use for nutritional intake and health outcomes across different income groups are shown in Table 6. We can see that the results for both income groups are consistent with the baseline results. There is a positive relationship between Internet use and HEI, DDS, overweight, obesity, chronic and health scores. However, in the low-income group, the regression coefficient of overweight is not significant. The regression coefficient of the variable Chronic in the high-income group is smaller than that in the low-income group, while the regression coefficient of the other variables is greater than that of the low-income group. Therefore, Internet use may have a greater impact on food consumption, nutritional outcomes, and health in high-income groups than in low-income groups.

**Table 6:** The heterogeneity analysis by income.

	HEI	DDS	Overweight	Obesity	Chronic	Health score
Low-income group						
Internet use	4.918*** (9.851)	1.231*** (10.420)	0.154 (0.917)	0.358*** (3.333)	0.759*** (2.853)	2.442*** (11.664)
Wald chi^2^	1165.83***	100363.52***	2275.99***	817.77***	126.54***	3048.09***
Observations	11,087	13,142	12,591	12,591	13,052	13,142
High-income group						
Internet use	9.600*** (12.524)	1.645*** (12.777)	0.388** (2.571)	0.393*** (3.555)	0.560** (2.352)	3.718*** (15.453)
Wald chi^2^	2360.37***	244113.28***	2473.74***	988.64***	581.13***	2644.37***
Observations	9,018	13,421	12,775	12,775	13,385	13,421

The fixed effects are used to estimate the results and the robust standard error deviation is in parentheses. ***p < 0.01; **p < 0.05; *p < 0.1.

## 4 Discussion

In the past few decades, Internet penetration in China has risen rapidly. By the end of 2016, the number of Internet users in China had reached 731 million, placing the country first in the world, with an average annual growth rate of more than 40 million (6.2%) (38). The rapid development of the Internet has changed people’s food consumption patterns and living habits (39). These changes have further affected the population’s food consumption, nutritional outcomes, and health status. As shown in Figure 2, China’s Internet user is comparable to the world average level. And China is the largest developing country in the world, and findings such as the effects of Internet use on nutritional intake and health outcomes in its rural area may have some implications to other developing countries. While the Internet use is also growing rapidly in developing countries such as India, Vietnam, and Thailand, and the quality of diet is improving, nutritional health is an important issue for all developing countries. Therefore, the results of this study may have important reference significance for policymakers not only in China but also in other developing countries. In addition, although there are big differences between China and European and American countries in terms of economic and social development level, dietary structure, etc., the influence channels of Internet use on nutritional intake and health outcomes may be consistent. In this study, the influence channels of diet quality and physical activity have been verified, which can also be used as reference for the research on Internet use and nutrition and health in developed countries.

This study found that Internet use significantly improved dietary quality, such as HEI and DDS, which is consistent with the findings of existing literature. For example, Xue et al. (12). found that Internet access significantly increased protein intake, which could be conducive to improving dietary quality. Ma and Jin found that there is a positive relationship between Internet use and a healthy diet. Cui et al. (40) argued that Internet use can improve family dietary quality like the Chinese diet balance index (DBI). In rural China, the reasons why Internet use can improve dietary quality may be mainly due to the following two points. First, the Internet is one of the main ways to obtain nutrition and health knowledge, especially in rural areas. Therefore, Internet use can improve dietary quality by improving dietary knowledge. Second, the rise of online shopping can help rural residents buy food that is not available in nearby markets, improving food availability. Therefore, Internet use may have a significant positive impact on dietary quality.

Another important finding of this study is that Internet use increases the risk of overweight and obesity among rural Chinese residents. This finding is consistent with the findings of Corneel et al. (41). They believe that Internet use increases the likelihood of overweight and obesity among Australian students because it increases the amount of time spent watching TV and being sedentary. However, our results are contrary to those of Chen and Liu (42), who concluded that Internet access increases the income of urban residents and thus reduces their risk of being overweight. This difference may be due to systematic differences in income structure, living habits, and dietary intake between rural and urban residents. Previous studies also found that the relationship between income and nutrition outcomes is opposite in rural and urban China (43, 44). We also found that one of the important reasons is that Internet use increased the risk of overweight and obesity was to reduce the frequency of physical activity among respondents. One interesting finding is that for young adults and females, Internet use will not increase their risk of overweight and obesity. It is possible that younger, younger, and female groups are more concerned about weight management than male and older age groups. It is worth noting that current articles on Internet use in developing countries mostly focus on urban areas and mainly study the positive effects of Internet use on food consumption and nutritional intake. Therefore, our findings are an important supplement to the existing literature.

This study finds that there is a positive relationship between Internet use and the self-assessment score of health, which is consistent with the results of Luo et al. (45). Besides, Ding et al. (46) also argue that the Internet is beneficial to the mental health of Chinese older adults. However, Ma and Sheng (47) find that Internet use may reduce duration and parent-adolescent communication, thus hurting their mental health. Therefore, the impact of Internet use on different countries and groups may varied. At the same time, we also find that Internet use increases the likelihood of chronic disease. It can be explained as the same as overweight and obesity, that is because Internet use reduces physical activities. Considering that these three variables of overweight, obesity, and chronic are more objective than Health score, we argue that Internet use improves diet quality, whereas it reduces physical activity, Internet use has a negative impact on the health of rural residents. Therefore, Internet use has both positive and negative effects on the nutrition and health of Chinese rural residents.

There are some limitations in this study. First, due to data limitations, we only used hypertension and diabetes to measure chronic diseases. But follow-up studies could use other chronic conditions to measure health, such as anemia or hyperlipidemia. In the past two decades, some chronic diseases such as overweight and obesity have grown rapidly in China, rising rapidly from the long-term lower than the world level to the higher than the world level. Second, the data in this study is from 2004 to 2015, although this includes the 10 years with the fastest development of the Internet in China, it would be preferable if subsequent studies could use panel data from more recent years.

## 5 Conclusion

A series of studies have discussed the effect of Internet use on food consumption, but the impact of Internet use on nutritional outcomes and health is little known, especially in developing countries such as China. Using multiple years of CHNS data and regression analysis, this study finds that Internet use improved dietary quality, such as HEI and DDS among Chinese rural residents. However, Internet use may increase the risk of overweight, obesity and health problems in the study regions. One important reason is that Internet use reduces their physical activity. The study also finds that the impact of Internet use on overweight and obesity is not significant among women and young people.

Given that the rapid increase of number of the Internet users and the changing lifestyle (e.g., e-commerce) in Chinese rural area, it is suggested that policymakers may seriously take its negative effects into account when promoting the development of the Internet. For instance, enlarge the investment in the Internet infrastructure while assign more physical facilities to the rural communities. Providing more dietary knowledge and cultivate the awareness of the nutritional self-care to the male and the relative older people when using the Internet. Insights and experiences from the study may also be valuable for the nutritional well-being in those developing regions in the digital era.

## Data availability statement

Publicly available datasets were analyzed in this study. This data can be found at: http://www.cpc.unc.edu/projects/china.

## Author contributions

ZD: Writing – original draft. JL: Writing – original draft, Conceptualization, Data curation, Formal analysis, Funding acquisition, Methodology, Project administration, Software, Supervision, Validation, Writing – review & editing. YH: Conceptualization, Supervision, Validation, Writing – review & editing. WL: Validation, Writing – review & editing.
